# Mixed Infection of Respiratory Tract in a Dog Caused by *Rhodotorula mucilaginosa* and *Trichosporon jirovecii*: A Case Report

**DOI:** 10.1007/s11046-017-0227-4

**Published:** 2017-12-01

**Authors:** Małgorzata J. Biegańska, Magdalena Rzewuska, Iwona Dąbrowska, Bożena Malewska-Biel, Magdalena Ostrzeszewicz, Bożena Dworecka-Kaszak

**Affiliations:** 1 Department of Preclinical Sciences, Warsaw University of Life Sciences, Warsaw, Poland; 2 Department of Small Animal Diseases with Clinic, Warsaw University of Life Sciences, Warsaw, Poland

**Keywords:** *Rhodotorula mucilaginosa*, *Trichosporon jirovecii*, *Bronchotracheitis*, Dog, Opportunistic mycosis

## Abstract

This report describes the isolation of two environmental fungi: *Rhodotorula mucilaginosa* and *Trichosporon jirovecii* accompanied by *Pseudomonas aeruginosa* and *Escherichia coli* from a dog with bronchotracheitis. All microorganisms were isolated routinely from a mucopurulent discharge, obtained during bronchoscopy from laryngotracheal area. The initial identification of yeasts was confirmed by API Candida and by molecular analysis of internal transcribed spacer region. Administered antimicrobial treatment with Marbofloxacin and Canizol has brought the improvement in the dogs’ health status. The final results of control mycological culture were negative. Most probably underlying hypothyroidism and the use of steroids were the factors predisposing this patient to opportunistic infection of mixed aetiology. As far as we are concerned, this is the first case of dogs’ respiratory tract infection caused by *R. mucilaginosa* and *T. jirovecii.*

## Introduction

*Rhodotorula* and *Trichosporon* yeasts belong to phylum *Basidiomycota*. Both fungi are widely distributed in the environment [1–3]. *Trichosporon* species were isolated from fresh water samples obtained from rivers and lakes, seawater, soil and decomposed wood. These fungi are also present in birds’ and mammals’ faeces [1, 3–6]. The members of *Trichosporon* genus can colonize gastrointestinal tract, oral and nasal cavity or upper part of respiratory tract [3]. Quite frequently *Trichosporon* species have been isolated from healthy skin, especially in the perigenital and perianal area [3]. *Rhodotorula* yeasts have been found in healthy cats [7], rhesus macaques [8], camels [9] and ostriches [10].

The genus *Trichosporon* was designated in 1865 by Beigel, as the organism causing hair infection of mild intensity, what became the basis for the creation of the genus name. Nowadays, when phenotypic identification of yeasts is supported by genetic analysis, the genus consists of multiple species arranged in four clades: Gracile/Brassicae, Porosum, Cutaneum and Ovoides [3, 11]. According to Colombo et al. [3], the genus includes a total of 50 species. Sixteen of them, belonging to first three mentioned clades, have clinical relevance [3]. Among them are *T. ashaii*, *T. inkin*, *T. jirovecii*, *T. ovoides*, *T. cutaneum* and others. The virulence factors shared by *Trichosporon* species are the ability to form biofilm and production of multiple enzymes (proteases, DNases, lipases), capable of destroying various components of host tissues. Moreover, one of the cell wall components—glucuronoxylomannan (GXM)—resembles GXM of *Cryptococcus neoformans* and may facilitate the dissemination within the host organism and inhibit phagocytic activity of monocytes or neutrophils, thus increasing the yeasts pathogenicity [1, 3, 12, 34].

*Rhodotorula* yeasts are also ubiquitous organisms, found in various ecosystems, e.g. seas, lakes and soil [13–15]. They are also isolated from birds and animals [4, 5, 7–9, 15]. All of *Rhodotorula* yeasts produce carotenoids, and their colonies develop a characteristic salmon pink colour [15, 16].

*Rhodotorula* genus encompasses eight species. Three of them including *R. mucilaginosa*, *R. glutinis* and *R. minuta* are supposed to be opportunistic, as they have been isolated from clinical infections in humans and other animal species [2, 11, 15, 17–21]. Krzyściak [22] have proved the presence of some of fungal virulence factors alike the production of extracellular enzymes in tested *Rhodotorula* strains. Some *R. mucilaginosa* strains had significant activities of phospholipase and aspartyl protease, while others have been producing proteolytic enzymes. Also some of them have revealed resistance to common antimycotic agents [17, 22]. Most of the tested strains were able to grow and to form the biofilm in serum in 37 °C, what is crucial in pathogenesis of fungal infections [22].

Additionally, *Rhodotorula* and *Trichosporon* fungi have been isolated from many unfavourable conditions, like hyperosmotic environments or located on high altitudes [13–15, 23, 24]. They were also found in water samples, obtained from the Baltic Sea at depths between 201 and 444 m [15, 25]. Some reports have noted their ability to decontaminate water and soil polluted by heavy metals or petroleum derivatives [26–28].

All metabolic properties of both genera allow them for easier adaptation to various conditions. At the same time, these adaptations make them capable of colonizing humans and other animal species [1, 3, 5–9]. The increasing number of clinical reports strongly suggests that some species of *Rhodotorula* and *Trichosporon* have the potential to infect humans [1–3, 29]. Additionally, there are references showing the pathogenicity of these yeasts for domestic and wild animals [18–21], which authorizes their classification as the opportunistic and emerging pathogens. In this work, we would like to present the case report, in which *Rhodotorula mucilaginosa* and *Trichosporon jirovecii* were isolated from the respiratory tract of a dog.

## A Case Report

A female dog, crossbreed, aged 10 years and 3 months, was submitted to the Department of Small Animal Diseases with Clinic, in Warsaw University of Life Sciences in November 2016. According to the medical history, the first clinical signs like cough followed by several incidences of dyspnoea, increased appetite and unwillingness to move were observed by the owner from March 2016. The previous treatment with steroids did not bring the clinical improvement. Due to the observed symptoms, hypothyroidism or heart problems were suspected. During the medical examination, there were no auscultatory changes observed in the lungs. Commissioned X-ray examination of the chest showed no perceptible changes in the lungs or heart. In contrast, blood biochemistry testing has confirmed the suspicion of hypothyroidism and showed elevated concentrations of liver enzymes. Other parameters were within normal limits. Oral administration of thyroid hormones together with withdrawal of steroids and proper diet was recommended. Applied treatment has resulted in a slight improvement in the health status, but in the next weeks incidences of cough intensified by the movement of animal have been observed several times per day. Subsequent laboratory tests carried out in January 2017, showed no significant abnormalities in the image and blood counts and most of the biochemical parameters were at normal level, including the concentrations of thyroid hormones. At the end of January 2017, due to persistent cough, occurring several times per day and aggravated by animal movements, the bronchoscopy was recommended and performed under general infusion anaesthesia. In this study, laryngeal oedema and mucopurulent discharge in laryngo-tracheal area, together with the first degree collapse of trachea and major bronchi, have been found. During the endoscopy, the specimens for cytopathologic and microbiologic examinations have been taken.

## Materials and Methods

Sample of laryngo-tracheal discharge obtained aseptically from dog’s trachea during the bronchoscopy was examined routinely for microorganisms. Clinical material was used to inoculate Schaedler broth medium, Columbia Agar with 5% of sheep blood (CA) and McConkey Agar (McC) (GRASO, Poland). All inoculated solid media were incubated in aerobic condition for 24 h in 37 °C. The Schaedler broth was incubated in anaerobic conditions in 37 °C for 48 h. Also direct microscopic slide stained with Gram method was prepared. After the incubation, bacterial growth was evaluated and the identification was done based on the phenotypic properties.

Clinical samples were also used for routine mycological examination with the use of Sabouraud Chloramphenicol Agar (bioMerieux, France) and Sabouraud Chloramphenicol Agar supplemented with 0,7% of cycloheximide. Inoculated media were incubated in 30 °C in aerobic conditions for 72 h, and the fungal growth was evaluated every 24 h. The primary identification was based on the morphology of the cells and the colonies. It was confirmed by the API Candida biochemical test (bioMerieux, France).

The antimicrobial susceptibility testing for isolated bacteria was performed on Mueller–Hinton Agar (GRASO, Poland) according to Kirby-Bauer disc diffusion method. Both isolated yeasts were also tested for antimycotic drug susceptibility, using the diffusion method performed according to the procedure recommended by the producer of MASTRING-S LAB 504/1 rings (Mast Diagnostics)

### Molecular Identification

To confirm the phenotypic identification of both isolated fungi, the sequencing of ITS1-5.8S-ITS2 regions was performed. The PCR amplification of the internal transcribed spacer ITS 1-5.8S-ITS2 ribosomal DNA region is widely used for identification of medically important yeasts and detection of dermatophytes in clinical specimens [30, 31]. The ITS region analysis is also suitable for interspecies differentiation and taxonomy studies of clinical, fungal isolates or environmental fungi, both yeast and mycelial species [6, 26, 30, 32].

Genomic DNA was extracted and purified using Genomic Mini AX Yeast (A&A Biotechnology, Poland) according to the manufacturer procedure. To amplify the ITS1-5.8S-ITS2 region, the conserved primers described previously by White et al. [33] were used. Briefly, each reaction was performed by the addition of 4.5 μl of the DNA and 0.75 μl of each primer (20 μM) to 15 μl of 2xPCR Master Mix Plus (A&A Biotechnology, Poland) and H_2_O in a total volume of 30 μl. The amplification was performed in Eppendorf Mastercycler thermocycler with the following profile: one initial cycle of denaturation for 3 min at 94 °C, followed by 34 cycles of 30 s at 93 °C, 30 s at 50 °C and 45 s of extension at 72 °C. The presence of specific PCR products of approximately 600 bp for *Rhodotorula* spp. and 500 bp for *Trichosporon* spp. was determined by electrophoresis in a 2% agarose gel containing ethidium bromide. To purify the rest of PCR products, a Clean-Up Concentrator kit (A&A Biotechnology, Poland) was used. The purified products were sequenced in outside company—Genomed, Poland. All sequences were compared with sequences available in GenBank using the BLASTN algorithm at National Center for Biotechnology Information (NCBI) and Centraalbureau voor Schimmelcultures (CBS) database using pairwise sequence alignment.

## Results

Multiple, gram negative rods and yeast-like cells were observed in direct microscopic slide. After 24 h of incubation in aerobic conditions, the two types of colonies were isolated on Columbia Blood Agar and MacConkey Media. The isolates were identified based on their phenotypic properties as *E. coli* (non- haemolytic strain) and *Pseudomonas aeruginosa*. There was no growth of anaerobic bacteria.

**Fig. 1:**
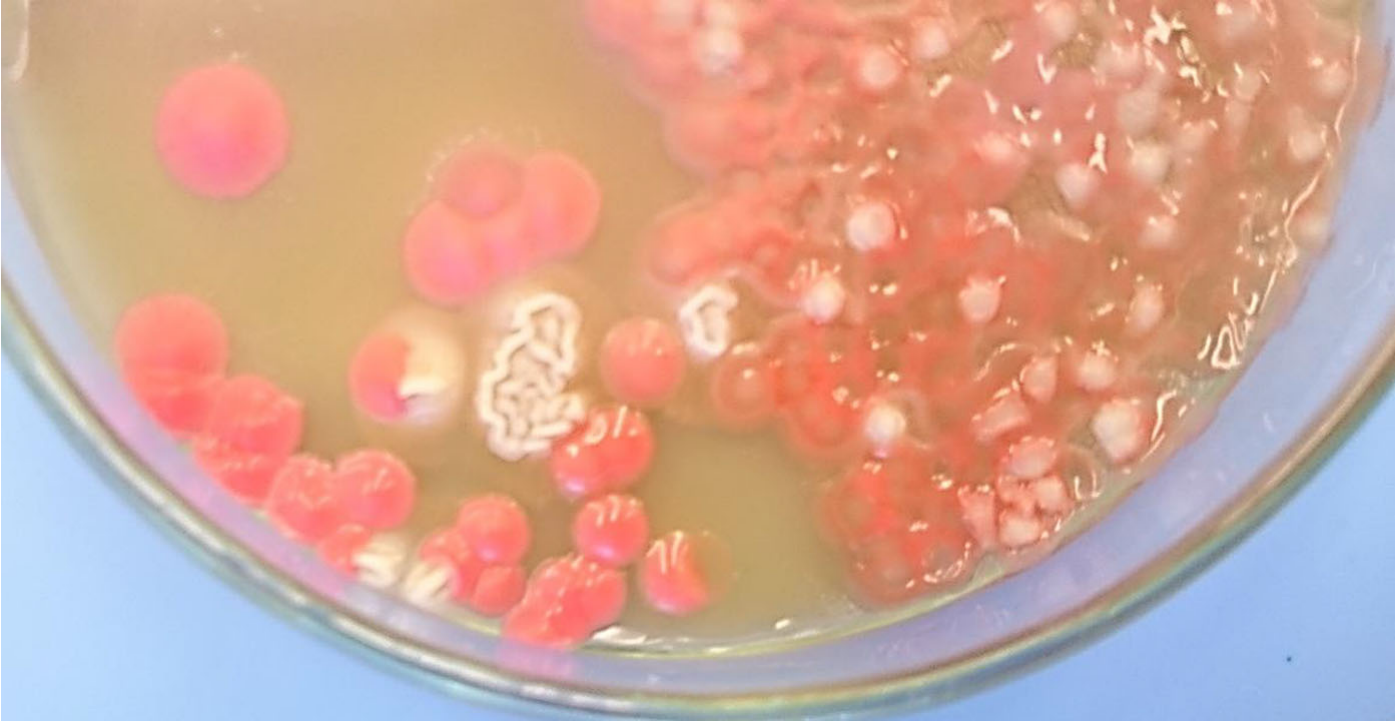
Abundant growth of yeasts’ colonies from the sample of bronchial mucopurulent discharge obtained from dog (SDA). Colony morphology typical for *Rhodotorulla* sp. and *Trichosporon* sp.

**Fig. 2:**
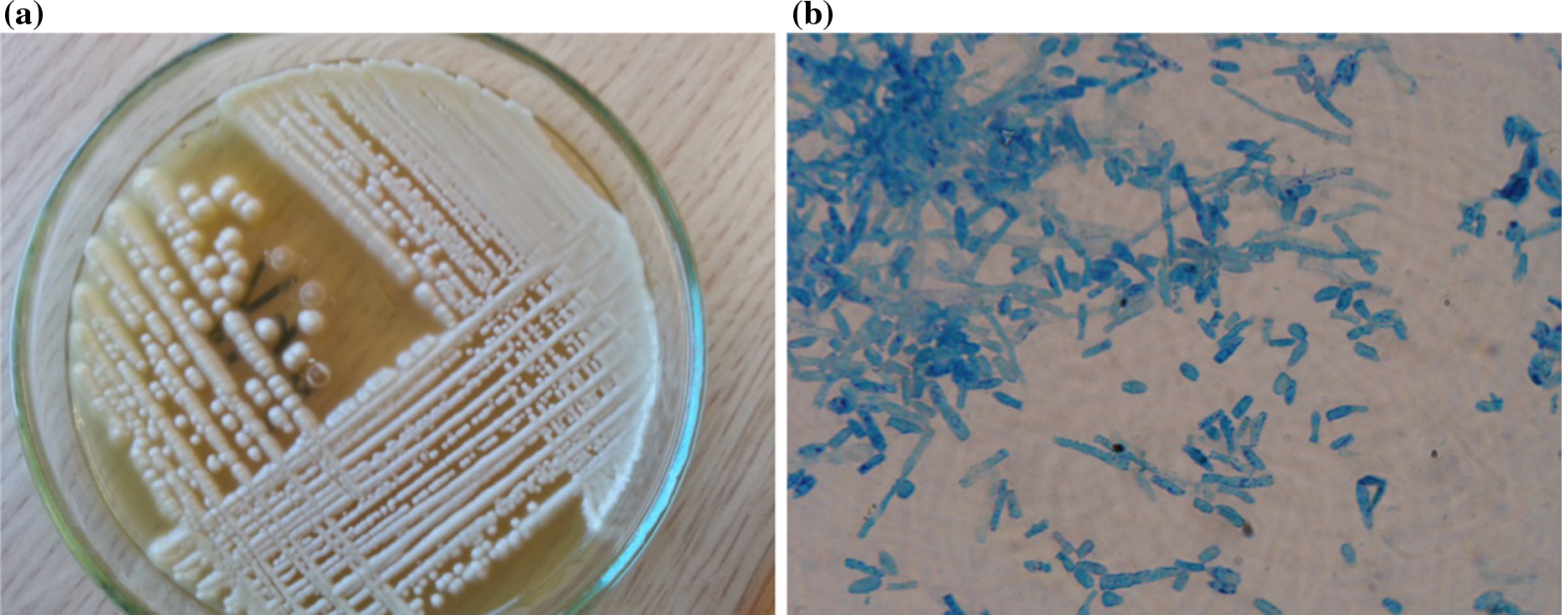
**a** Isolated colonies of *Trichosporon* sp. **b** Microscopic slide prepared from colony stained with methylene blue. Typical blastospores of *Trichosporon* sp. ×1000

After 48 h of aerobic incubation of Sabouraud Chloramphenicol Agar in 30 °C, multiple smooth, orange-pink, shiny colonies together with very small, white, dull colonies were observed (Fig. 1). Additional 24 h of incubation allowed for better growth of white colonies (Fig. 2a). Microscopic slides prepared from both types of colonies, stained with methylene blue, have showed the presence of oval-shaped, yeast-like cells in pink colony and elongated blastospores in white, folded colony (Fig. 2b). Based on the morphology, the isolates were identified as *Rhodotorula* sp. and *Trichosporon* sp. The identification of both isolates was confirmed by API Candida biochemical tests (bioMerieux).

Additional identification of both strains to the species level was done by molecular methods. Extracted and purified DNA was used to amplify the ITS1-5.8S-ITS2 rRNA regions by the PCR protocol described above. These fragments are recommended for the phylogenetical analysis of fungi [33]. The obtained sequences were compared to the fungal sequences available in GenBank (NCBI) and to CBS databases. The comparison of first sequence has revealed a 99% similarity (95% of cover) with the sequence of *R. mucilaginosa* strain IIFCSW-B2 (Accession number: KY218730.1). The second sequence has showed 99% identity (95% of cover) with the sequence of *T. jirovecii* strain ATCC 34499 (Accession number: HM802131.1).

Antimicrobial testing results showed that both bacterial isolates were multiresistant. They were resistant to most antimicrobials, which could be applied in the treatment of respiratory infections in dogs. Because of previously observed liver damage, the only possibility to treat bacterial infection was the use of marbofloxacin.

**Fig. 3:**
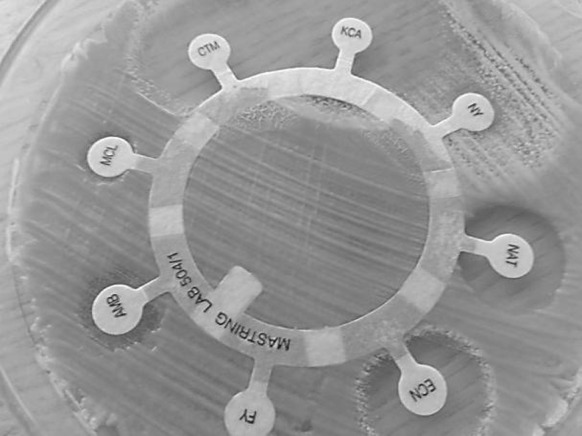
Drug susceptibility testing for both yeasts’ strains (MASTRING-S LAB 504/1; Mast Diagnostics)

In antifungal susceptibility testing (Fig. 3), after 24 and 48 h of incubation, the resistance of both isolates to Flucytosine, Amphotericin B and Miconazole was observed, while both fungi were susceptible to Econazole, Clotrimazole and Ketoconazole, as well as to Natamycin. All macrolide polyene antifungals were excluded from treatment: Amphotericin B due to the resistance noted during the tests and both Natamycin and Nystatin because of their limited absorption from the gastrointestinal tract. Formulations of these two compounds available in Poland are used mostly topically as the creams, lozenges or vaginal tablets. Finally, based on the noted high activity against both fungal isolates, oral administration of Canizol (Ketoconazole) in the dose of 100 mg two times per day was prescribed.

Administered antimicrobial treatment has brought the improvement in the patients’ health status, and the result of control mycological culture was negative.

## Discussion

Before the 1980s, the medical or veterinary reports mentioning *Rhodotorula* and *Trichosporon* as the aetiological agents of human or animals mycoses were sparse [1, 2, 15, 34, 35]. During the last decade, the increasing number of articles reporting clinical cases of infections caused by these yeasts has been published [1, 2, 11, 15, 34, 35]. One of the most prevalent circumstances that increases the risk of invasive fungal infection is the use of central venous catheters (CVCs) and other indwelling medical devices. The species of both genera are able to form biofilm on various materials used for their construction, e.g. polystyrene [1, 12]. Similarly, *Rhodotorula* is able to colonize shower curtains, tooth brushes, dialysis equipment, endoscopes [15] and hydratation sport tanks or drinking bottles [36]. The formation of biofilm contributes on persistency of infection, being in fact the permanent source of fungal cells. It also significantly raises the resistance to antifungal agents and restrains the treatment possibilities.

Predominant type of human infections caused by two described yeast genera is fungemia connected with the necessary use of central venous catheters. The most prevalent species of *Rhodotorula* are: *R. mucilaginosa*, *R. glutinis* and *R. minuta*. In the vast majority of these cases, there were underlying diseases predisposing to development of mycoses, e.g. haematological malignancies, cancer, chronic renal failure requiring continuous ambulatory peritoneal dialysis (CAPD), gastrointestinal diseases or AIDS. In the group with high risk of yeasts infections were the individuals undergoing long-term therapy with antibiotics or steroids, transplant receivers and neonates [2, 11, 15, 34]. Besides the infections via CVC, other mycoses caused by *Rhodotorula* yeasts have been noted. Some of them have developed superficially e.g. dermatitis, onychomycosis, oral infections and ulcers. The others were localized in the internal organs—the cases of meningitis, endophthalmitis, peritonitis, endocarditis, corneal or bone infections have been described [2, 11, 15, 29].

The *Trichosporon* species may be involved in the aetiology of superficial infections and white piedra, as well as in mycoses located deeply in host organism, such as hypersensitivity pneumonitis or invasive trichosporonosis [1, 3, 34]. Most frequently causative agent of superficial and invasive infections in humans are: *T. inkin*, *T. cutaneum T. ovoides* and *T. loubierii*, followed by *T. ashaii*, *T. mucoides* and *T. asteroides* [1, 3, 34, 35]. The most important reservoirs of microorganisms are gastrointestinal and respiratory tracts colonized by *Trichosporon* species [35]. Moreover, in invasive trichosporonosis, the vast majority of patients shared some predisposing factors with individuals suffering from invasive rhodotorulosis. In the group at high risk of severe deep-seated *Trichosporon* infections are patients with haematological neoplasms and other cancers, especially those requiring the use of intravascular catheters or peritoneal dialysis, in which the colonized skin might be the additional source of fungi. On the contrary, superficial infections and hypersensitivity pneumonia are found more frequently in immunocompetent hosts, but the sources of infections in these cases are still discussed. The routes of infections are also unclear, but it is suggested that poor hygiene, close contact with contaminated water or infected individuals may increase the risk of trichosporonosis [3, 34, 35].

Despite many papers describing humans’ infections caused by *Trichosporon* and *Rhodotorula*, there are only few reports about mycoses caused by these fungi in other animal species. One of the first reports describing animal infections caused by *Rhodotorula* was published in 1970 by Beemer et al. [37] and in 1980 by Aruo [38]. The authors presented data about skin rhodotorulosis in chickens [37, 38]. In 1992, Bourdeau et al. [39] described the suspicion of *Rhodotorula* dermatomycosis in cat with FeLV and FIV infection. Kadota with co-workers [21] reported the case of granulomatous epididymitis in a dog, in which *Rhodotorula glutinis* was the aetiological agent. Agnetti with team [40] presented the case of a dog with generalized pyoderma and alopecia-crusted lesions, observed on the head, the ears, back and chest. During the laboratory tests, the authors have isolated *R. glutinis* and confirmed the identification by API ID32C test (bioMeriux) and PCR. The treatment with ketoconazole was successful and led to negative results of control cultures [40]. Also there is the clinical report announcing the mycosis caused by *R. mucilaginosa* in female sea lion [20]. The probable source of infection could have been the water contaminated by *Rhodotorula* yeasts, which have been found in seawater [15] Additionally, in captive or zoo animals, the important factors predisposing to mycosis are various stresses connected with captivity.

*Trichosporon* infections in animals seems to be rare. Dörlemann et al. [41] described multiple invasive infections caused by *Trichosporon beigelii* in the group of 56 laboratory rats. The animals were specially prepared to drug trials for the treatment of pneumonia caused by *Pneumocystis carinii*. They received abnormal, low-protein diet and corticosteroid treatment; thus, they were immunosuppressed. The source of invasive *T. beigelii* infection was contaminated inoculum of *Pneumocystis*, prepared from the lung tissue of rats with pneumocystosis.

In farm animals, the most frequent infections caused by the members of both described genera and other fungi are mastitis cases [18, 19, 42]. In milk samples obtained in Poland from cows with mastitis, we have isolated *Rhodotorula* yeasts together with different species of *Candida* [18]. Comparable findings have noted Wawron and his team [19]. Fungi were isolated from milk samples obtained from *mastitis* cases in 7.07% of all aetiological agents. Among them were: *Candida*, *Trichosporon*, *Rhodotorula* and *Cryptococcus* yeasts. Nearly the same genera were isolated in Brasil, in Sao Paulo State during the survey done by Costa et al. [42]. Other bovine infections described in literature were cases of mixed fungal infections accompanying mixed parasitic infestations of ear canals. In 11% of positive mycological cultures, Duarte et al. [43] have isolated *Rhodotorula* yeasts.

All mentioned medical reports show that yeasts from both genera have the wide range of adaptation abilities, what may predispose them to colonize the new ecological niches or hosts, both animals and humans. Despite the small number of published clinical veterinary reports, all presented literature data show that medical conditions occurring in animals are comparable to those in humans and can be the factors favouring opportunistic mycoses.

Our work seems to be the first case report describing *bronchotracheitis* of mixed yeasts and bacterial aetiology in dog. In this case, the factors predisposing the animal for the development of opportunistic mycosis were hypothyroidism, age of the animal and the steroid treatment. The fact that clinical specimen was obtained from the trachea during aseptical bronchoscopy procedure may exclude the suspicion of contamination. Thus, our belief that the aetiological agents of *bronchotracheitis* were *R. mucilaginosa* and *T. jirovecii* followed by *P. aeruginosa* can be confirmed. Moreover, in our study, *R. mucilaginosa* and *T. jirovecii* are described for the first time as the aetiological agents of *bronchotracheitis* in dog.
